# Orexin Receptor Blockade-Induced Sleep Preserves the Ability to Wake in the Presence of Threat in Mice

**DOI:** 10.3389/fnbeh.2018.00327

**Published:** 2019-01-08

**Authors:** Shouhei Iwakawa, Yuichi Kanmura, Tomoyuki Kuwaki

**Affiliations:** ^1^Department of Physiology, Graduate School of Medical and Dental Sciences, Kagoshima University, Kagoshima, Japan; ^2^Department of Anesthesiology and Critical Care Medicine, Graduate School of Medical and Dental Sciences, Kagoshima University, Kagoshima, Japan

**Keywords:** orexin, hypocretin, hypnotics, dual orexin receptor antagonist, triazolam, aversive stimuli

## Abstract

Retention of the ability to wake from sleep in response to dangerous situations is an ideal characteristic of safe hypnotics. We studied the effects of a dual orexin receptor antagonist-22 (DORA-22) and the GABA-A receptor modulator, triazolam, on the ability to wake in response to aversive stimuli. We examined four modalities of sensory inputs, namely, auditory (ultrasonic sound), vestibular (trembling), olfactory (predator odor), and autonomic (hypoxia) stimuli. When the mice fell asleep, one of the four stimuli was applied for 30 s. In the case of auditory stimulation, latency to arousal following vehicle, DORA-22, and triazolam administration was 3.0 (2.0–3.8), 3.5 (2.0–6.5), and 161 (117–267) s (median and 25–75 percentile in the parentheses, *n* = 8), respectively. Latency to return to sleep after arousal was 148 (95–183), 70 (43–98), and 60 (52–69) s, respectively. Similar results were obtained for vestibular and olfactory stimulation. During the hypoxic stimulation, latencies for arousal and returning to sleep were not significantly different among the groups. The findings of this study are consistent with the distinct mechanisms of these sleep promoting therapies; GABA-A receptor activation by triazolam is thought to induce widespread central nervous system (CNS) suppression while DORA-22 more specifically targets sleep/wake pathways through orexin receptor antagonism. These data support the notion that DORA-22 preserves the ability to wake in response to aversive and consciousness-inducing sensory stimuli, regardless of modality, while remaining effective in the absence of threat. This study provides a unique and important safety evaluation of the potential for certain hypnotics.

## Introduction

Although living in the modern world allows us to encounter dangerous situations far less frequently than what wild animals may encounter, it is still important for humans to be able to awaken quickly during natural disasters such as earthquakes, volcanic explosions, and fires. Stress and anxiety resulting from worrying about sleeping through these occurrences may lead to conditions such as insomnia.

Even during sleep, the brain continuously processes sensory information. This has been demonstrated by brainstem auditory evoked potential recordings (Perrin et al., 1999) and neuroimaging (Portas et al., 2000). The threshold required for the sensory input to reach the cerebral cortex, however, is higher during sleep than when awake due to thalamic sensory gating (McCormick and Bal, 1994). Central nervous system (CNS) depressants, such as benzodiazepines, also affect the threshold required in order for sensory input to evoke arousal. For example, the short-acting benzodiazepine triazolam impaired the ability in sleeping humans to wake up upon exposure to a loud fire alarm (Johnson et al., 1987).

The orexin/hypocretin-signaling pathway was discovered in 1998 (de Lecea et al., 1998; Sakurai et al., 1998) and plays an important role in regulating arousal and sleep (Sakurai, 2007) as well as vigilance state-dependent changes in autonomic functions (Kuwaki, 2015; Carrive and Kuwaki, 2016). Dual orexin receptor antagonists (DORAs) that block orexin receptors 1 and 2 have recently been developed and promote sleep through a decrease in arousal signaling (Gotter et al., 2014). GABA-A modulators, the most widely used hypnotic (Roehrs and Roth, 2000; Wang and Liu, 2016), and DORAs have different sleep promoting mechanisms, so it stands to reason that their effect on sensory-input induced arousal may also be different.

Tannenbaum et al. (2014) previously showed that one of the DORAs, DORA-22, did not impair the ability to wake in response to emotionally salient acoustic stimuli in dogs. In their study, the authors used an acoustic tone classically conditioned to be associated with a food reward. In almost all of the trials, DORA-22-treated dogs woke up in response to the salient positive stimulus but not to the neutral stimulus in a similar way to when they received no drug. The same group of authors later showed similar results with monkeys (Tannenbaum et al., 2016). Unfortunately, however, the authors mentioned only tested positive but not negative emotion-associated stimulus. Another weak point of their studies was that they used cue-conditioned test paradigm but not innate salient stimuli. Therefore, possible effect of DORA-22 on sensory processing circuit is still an open question even though it may not affect memory retrieval process.

The purpose of the present study was to examine the possible effects of a DORA-22, on negative valence stimuli-induced arousal which is independent from learning and memory, and compare them with GABA-A receptor modulators, eszopiclone and triazolam. We also analyzed the latency to return to sleep after the stimuli ceased in order to evaluate any possible retention of sleep promoting effects from the drugs.

## Materials and Methods

### Animals

Experiments were conducted on male C57BL/6 mice (25–35 g, Clea Japan). Animals were maintained under normal laboratory conditions (controlled 23°C temperature and food/water *ad libitum*) under a regular 12-h light/dark cycle (19:00 lights off and 07:00 lights on). All experiments were performed during the dark phase when nocturnal mice are most active. Experiments were performed in accordance with the guidelines outlined by the Physiological Society of Japan (2015) and were approved by the Experimental Animal Research Committee of Kagoshima University (MD16051).

### Compounds

All pharmacological agents were diluted in 20% d-alpha tocopherol polyethylene glycol 1,000 succinate (Vitamin E-TPGS) vehicle to a dose volume of 0.1 ml/10 g, and were administered orally using standard stainless steel gavage needles affixed to a 1 ml syringe (p.o.). Hypnotics tested included a DORA-22 (100 mg/kg; Gotter et al., 2014; a kind gift from Merck & Co., Inc., Kenilworth, NJ, USA), triazolam (1.25 mg/kg; Sigma-Aldrich Corporation, St. Louis, MO, USA), and eszopiclone (15 mg/kg; Carbosynth Ltd., Compton, Berkshire, UK). Doses were determined according to previously published articles (Gotter et al., 2014) and clinical dosage information for humans (10 mg for suvorexant, a derivative of DORA-22, 0.125 mg for triazolam, and 1 mg for eszopiclone).

All mice received treatment with all drugs/vehicle in randomized order. An interval between administrations of at least 3 days was used according to the previous article (Winrow et al., 2012) to prevent any possible influences of repeated procedures and residual drug effects. Half-life off-rate of DORA-22 to orexin 2 receptor binding was reported to be 37.8 min (Gotter et al., 2013) and no next-day effects was reported at least in monkeys (Gotter et al., 2013).

### Sleep Recordings

Under isoflurane (1.5%–2.0%, inhalation through face mask) anesthesia, electrodes were implanted for EEG/EMG recording. Two holes were drilled in the skull, and the arms of the electrode for the EEG were implanted at sites approximately 1.5 mm lateral to the Bregma. EMG recording wires made of stainless steel (Cooner Wire, Chatsworth, CA, USA) were inserted into the neck muscles bilaterally. Each electrode was fixed rigidly to the skull with dental cement. After surgery, mice were given an antibiotic, penicillin G (40,000 U kg^−1^), and an analgesic (buprenorphine, 0.05 mg kg^−1^). Animals were individually housed and allowed to recover for at least 7 days. The implanted electrode of each mouse was connected to a cable for signal output. Signals were amplified (AVH-11, Nihon Kohden, Tokyo, Japan) and digitally recorded on a computer with signal processing software (Chart, ADInstruments Inc., Bella Vista, NSW, Australia). Sleep stages were judged according to the method previously published (Nakamura et al., 2003). In brief, wakefulness was defined by a high frequency (8–30 Hz) low-amplitude EEG with a high EMG tone. Slow wave sleep (SWS) was defined by a low-frequency (0.25–4 Hz) high-amplitude EEG. Non-SWS sleep or rapid eye movement (REM) sleep was defined by a mixed-frequency (4–8 and 8–30 Hz) low amplitude EEG associated with weak or absent EMG activity.

### Test for Sleep-Promoting Effects of the Hypnotics

Before the aversive stimuli-experiments, the length and magnitude of sleep-promoting effects of the hypnotics were tested in our experimental setting without any stimulation. Mice (*n* = 5) in their home cage were connected to the cable for EEG/EMG measurement at 19:00 when the dark phase begins. After 1 h of baseline measurement, either vehicle, DORA-22, triazolam, or eszopiclone was orally administered to the mice. Mice eventually received all four drugs in a random order. The sleep-wake rhythm was measured until 02:00 (6 h after administration). Night dosing was selected because in nocturnal mice the baseline time spent sleeping is smaller than it is in daytime and thus any sleep-promoting effects are easier to observe. Sleep time was calculated for every 30 min. Mice’s natural sleep is not continuous as humans but relatively short repetition of sleep and wake. Therefore, we thought that latency to arousal by aversive stimuli and latency to return to sleep should be evaluated in comparison with natural sleep/awake duration. For this purpose, we calculated episode duration of sleep and awake in this (without stimulation) experiment.

### Aversive Stimuli-Induced Arousal Testing

Stimulation experiments were conducted between 21:00 and 24:00, which correspond 1–4 h period after the drug administration, in the different sets of the animals to the above-stated without stimulation group. When the mouse fell asleep for more than 1 min and in SWS, one of the stimuli (see below) was applied and any possible effects of the stimulus on the sleep-wake cycle were observed for 30 min. The same stimulus was applied 2–3 times in one animal during the experimental period of 3 h and the average value was used as the representative value for the experiment. Only one type of stimulus was tested over the course of one experimental night.

The following four aversive stimuli were tested using different set of the animals. First, for auditory stimulation, ultrasonic sound (25 kHz, 100 dB, 0.5 s × 7 times, interval 4.5 s; Moriya et al., 2018) was applied from a position 20 cm above the sleeping mouse (*n* = 8). Ultrasonic sound was generated by PET-AGREE (apparatus used for training pets; K2 Enterprises, NY, USA). Second, for vestibular stimulation, trembling (180 rpm) was applied to a measuring cage containing a mouse (*n* = 8) for 30 s. Trembling was done by a shaker (mini-shaker PSU-2T; WakenBtech, Kyoto, Japan) on which the measuring cage was placed. Third, for olfactory stimulation, a cotton swab containing 10 μL of TMT (2,4,5-trimethyl-3-thiazoline, a predator odor which is extracted from fox feces) was placed at the distance of 1 cm from the tip of the mouse’s nose (*n* = 10) for 30 s (Tashiro et al., 2016). Fourth, for hypoxic stimulation, 10% O_2_ gas (1,000 ml/min) was introduced into a gas tight chamber (750 ml) in which the mouse (*n* = 8) was placed. Oxygen concentration in the chamber was monitored (model JKO-25LJ II CM, JIKCO, Tokyo, Japan) at the output port. Oxygen concentration in the chamber became 10% within 120 s, was maintained there for 180 s, and then returned to the normal room air concentration (21% O_2_).

For the sleep state analysis, we calculated the latency to wake up from the aversive stimulus and latency to return to sleep after arousal. Every mouse was tested for the same aversive stimulus after receiving vehicle, DORA-22, and triazolam.

### Statistical Analysis

In the test for the sleep promoting effects of the hypnotics, data were expressed as mean ± SEM. Statistical comparisons were performed using repeated measure ANOVA followed by Tukey’s multiple comparisons test. In the stimuli-induced arousal testing, data were expressed as median and 25–75 percentile because data were not normally distributed (see “Results” section) and non-parametric statistics were more suitable. Statistical comparisons were performed using the Friedman test, a repeated measure nonparametric multiple comparisons test. When appropriate, it was followed by Dunn’s *post hoc* test. All statistics were calculated using Prism6 software (GraphPad Software, Inc.). Differences were considered significant at *p* < 0.05.

## Results

### Sleep Time Without Aversive Stimuli

**Figure 1:**
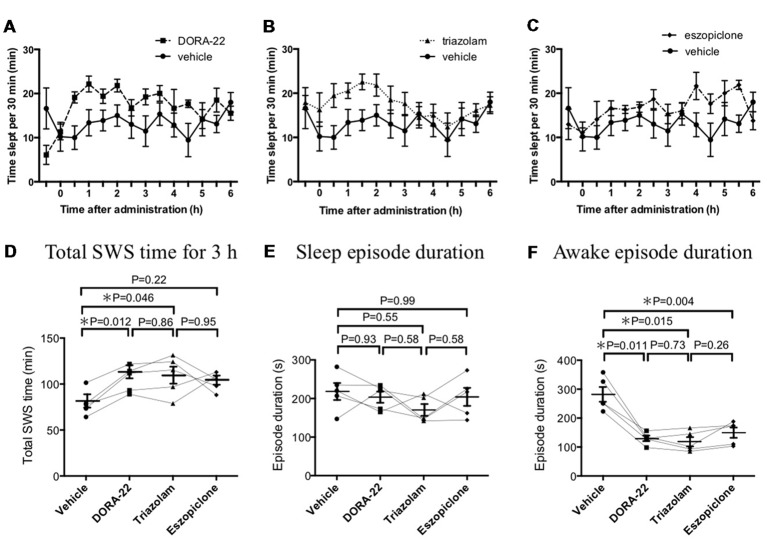
Effects of dual orexin receptor antagonist-22 (DORA-22), triazolam, and eszopiclone on sleep time and sleep architecture. Time slept per every 30 min is shown for **(A)** DORA-22, **(B)** triazolam, and **(C)** eszopiclone for 6 h after p.o. administration of the drugs. The same values for the vehicle are shown repeatedly for comparison purposes. Each animal received DORA, triazolam, eszopiclone, and vehicle in a randomized order on spaced days. Data are shown as Mean ± SEM. **(D)** Comparison among the four treatments (*F*_(3,12)_ = 5.101, *p* = 0.017) revealed that DORA-22 and triazolam, but not eszopiclone, significantly increased total sleep time during a 3-h period starting at 1 h after administration. These drugs did not affect sleep episode duration (*F*_(3,12)_ = 0.906, *p* = 0.467; **E**) but did decrease awake episode duration (*F*_(3,12)_ = 33.0, *p* < 0.001; **F**). In **(D–F)**, data from the same animal are connected with lines to show possible interactions between drugs in individual mice. Horizontal lines indicate mean value for each treatment. Statistical results using repeated measure ANOVA followed by Tukey’s multiple comparisons test are indicated in the graph.

Before the aversive stimuli-experiments, the length and magnitude of any sleep-promoting effects from the various hypnotics were tested in our experimental setting without any stimulation (Figure 1). During the 6 h of the observation period, mice spent 160 ± 11 min, 221 ± 6 min, 210 ± 13 min, and 209 ± 7 min sleeping (SWS and REM sleep) under the effects of either vehicle, DORA-22, triazolam, or eszopiclone, respectively. Although all three drugs seemingly had a sleep promoting effect, the detailed characteristics were different. As to SWS duration, DORA-22 (206 ± 5 min, *p* = 0.006, *n* = 5, Tukey’s multiple comparison test) and triazolam (209 ± 13 min, *p* = 0.016) significantly increased and eszopiclone (205 ± 7 min, *p* = 0.057) tended to increase as compared to vehicle (154 ± 10 min). While on REM sleep, DORA-22 (15.3 ± 1.7 min, *p* = 0.033) significantly increased, triazolam (1.5 ± 0.2 min, *p* = 0.089) tended to decrease, and eszopiclone (3.9 ± 0.7 min, *p* = 0.784) showed no effect as compared to vehicle (5.1 ± 0.9 min). In addition, effect of eszopiclone appeared to have a later onset than DORA-22 and triazolam (compare Figures 1A–C). Since the main purpose of this study was to compare aversive stimuli-evoked responses among the hypnotics, we thought similar magnitude and similar time course of sleep-promoting effect of hypnotics would be desirable. From this consideration, we selected the 3 h starting from 1 h after the injection until 4 h after the injection for statistical analysis. In addition, we focused on SWS since duration of REM sleep was too short to evaluate aversive stimuli-evoked responses. Comparison among the 4 treatments by repeated measure ANOVA (*F*_(3,12)_ = 4.944, *p* = 0.018) and subsequent multiple comparison with Tukey’s test revealed that DORA-22 (*p* = 0.012) and triazolam (*p* = 0.046), but not eszopiclone (*p* = 0.22), significantly increased total SWS time during a 3-h period as compared to vehicle treatment (Figure 1D). These drugs did not affect sleep episode duration (*F*_(3,12)_ = 0.906, *p* = 0.467; Figure 1E) but did decrease awake episode duration (*F*_(3,12)_ = 33.0, *p* < 0.001; Figure 1F).

Thus, we confirmed that DORA-22 and triazolam had similar sleep promoting effects over a similar time course for the selected dosages. From these results, we decided to compare vehicle, DORA-22, and triazolam, but not eszopiclone, in the next step of aversive stimuli-induced arousal testing. The testing took place during the 1–4 h period after the drug injection because the lag period for drug absorption and distribution appeared to be approximately 1 h. The confirmation period required to define sleep before stimulation was performed was set as 60 s because each sleep episode typically lasted for approximately 200 s (Figure 1E).

### Aversive Stimuli-Induced Arousal and Return to Sleep After the Cessation of the Stimuli

Next, we examined whether the animal was able to promptly wake up from sleep induced by DORA-22 and triazolam in response to aversive stimuli. We also examined the latency to return to sleep after arousal. Latency to arousal and latency to return to sleep in drug-treated groups were compared with those in the vehicle-treated “natural” sleep group.

**Figure 2:**
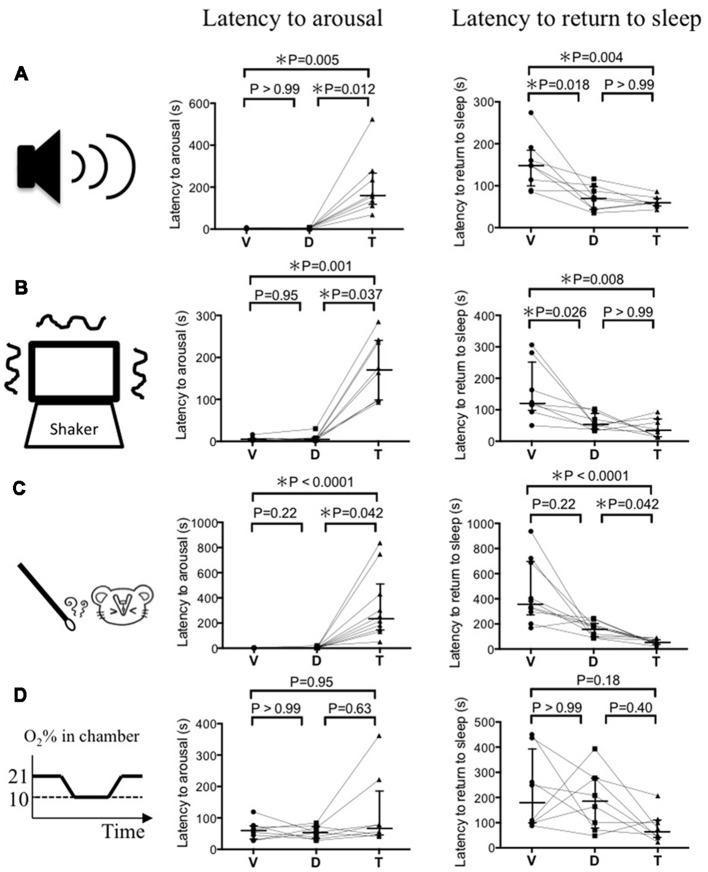
Latency to arousal and latency to return to sleep induced by four modalities of sensory stimulation. **(A)** Auditory stimulus by ultrasonic sound (*n* = 8). **(B)** Vestibular stimulus by cage shaking (*n* = 8). **(C)** Olfactory stimulus by a predator odor (*n* = 10). **(D)** Autonomic stimulus by hypoxia (*n* = 8). Each animal received vehicle (V), DORA-22 (D), and triazolam (T) in a randomized order on spaced days and were tested for one of the stimuli 2–3 times. The value from those tests were averaged and are represented as a single dot. Data from the same animal are connected with lines to show possible drug interactions within an individual animal. Horizontal lines indicate, from top to bottom, 75 percentile, median, and 25 percentile, respectively. Statistical results using the Friedman test, a repeated measure nonparametric multiple comparisons test, are indicated in the graph. Note that latency to arousal in DORA-22 treated mice is not different from those in vehicle treated mice and significantly shorter than those in triazolam treated mice, with the exception of hypoxia. Also note that latency to return to sleep in DORA-22 treated mice is significantly shorter than those in vehicle treated mice and not different from those in triazolam treated mice at least for auditory and vestibular stimuli.

For auditory stimulation (Figure 2A), the latency to arousal following vehicle, DORA-22, and triazolam administration was 3.0 (2.0–3.8), 3.5 (2.0–6.5), and 161 (117–267) s (median and 25–75 percentile in the parentheses, *n* = 8), respectively. After mice received the vehicle and DORA-22, they woke up during the stimulation period of 30 s but after triazolam mice woke up after secession of the stimulus. Latency to return to sleep after arousal was 148 (95–183), 70 (43–98), and 60 (52–69) s, respectively for vehicle, DORA-22, and triazolam. Returning to sleep was never observed during the stimulus period in all the drug treatments. Statistical analysis using the Friedman test and Dunn’s *post hoc* test showed that the latency to arousal was significantly prolonged by triazolam (*p* = 0.005) but not by DORA-22 (*p* > 0.99) when compared to vehicle. Latency to return to sleep was significantly shorter in DORA-22 (*p* = 0.018) and triazolam (*p* = 0.004) when compared to vehicle and there was no significant difference between DORA-22 and triazolam (*p* > 0.99). Similar results were obtained for vestibular stimulation (Figure 2B, *n* = 8) and olfactory stimulation (Figure 2C, *n* = 10). The only exception was that there was no significant difference between latency to return to sleep in the vehicle treatment and DORA-22 treatment (*p* = 0.22) after olfactory stimulation. This is probably because one out of 10 animals showed a longer latency to return to sleep under DORA-22 than they did with vehicle.

In contrast to the above-mentioned auditory, vestibular, and olfactory stimulation, there were no significant differences in the latencies to arousal and return to sleep among vehicle, DORA-22, and triazolam when hypoxia was used as a stimulus (Figure 2D, *n* = 8). There was a time lag of about 120 s before O_2_ concentration in the chamber reached 10% and most animals woke up within that 120 s. For example, the O_2_ concentration in the chamber when the mice woke up from sleep with vehicle, DORA-22, and triazolam was 16.3 (15.4–18.4), 16.4 (15.0–17.9), and 15.6 (14.4–17.6) % (median and 25–75 percentile in the parentheses), respectively. There was no significant difference among the drugs. Some animals re-slept even when the hypoxic O_2_ concentration of 10% continued. Out of the eight animals undergoing the hypoxic stimulation, the number that re-slept under the vehicle, DORA-22, and triazolam conditions were 5, 5, and 7, respectively. A chi-square test revealed no statistical differences (*p* = 0.446) among the treatments.

## Discussion

This study demonstrates that DORA-22 (100 mg/kg) and triazolam (1.25 mg/kg) had similar sleep promoting effects (30%–40% increase in SWS time as compared to vehicle) in a similar time course (approximately 4 h after oral administration) in mice. During this period, aversive stimuli-induced arousal and the return to sleep after arousal were examined. As expected, auditory stimulus-induced arousal was delayed significantly in the triazolam treatment (*p* = 0.005) but not in the DORA-22 treatment (*p* > 0.99) when compared to the vehicle treatment. Even though the DORA-22 treatment showed a short latency to wake up, the sleep-promoting effect of DORA-22 seemed to remain because the latency to return to sleep after arousal was significantly shorter than vehicle treatment and not different from triazolam. Similar results were obtained for vestibular and olfactory stimuli-induced arousal and return to sleep. In contrast, hypoxic stimulus-induced arousal and return to sleep were not different among groups.

We had expected that eszopiclone (15 mg/kg) would show a sleep promoting effect similar to triazolam and DORA-22 because eszopiclone as well as triazolam and suvorexant (another DORA) are effective clinically in humans (Matheson and Hainer, 2017). In a preliminary experiment, we tried a higher dose of eszopiclone (100 mg/kg, *n* = 2) but the result was similar to the selected dose of 15 mg/kg. Gotter et al. (2014) reported that the sleep-promoting effects of eszopiclone are highly species specific. In their study, they showed that treatment with eszopiclone resulted in consistent effects in rats and rhesus monkeys, variable effects in mice (60 mg/kg), and paradoxical hyperarousal in dogs. The reason behinds the differences in species has not yet been revealed, but the authors speculated that possible differences in the GABAergic pathways in the brains of these species may be the cause. It may be interesting to point out that activation of extrasynaptic GABA-A receptor in the pontine reticular formation promotes wakefulness (Vanini and Baghdoyan, 2013). In any case, we stopped further experimentation using eszopiclone because our main purpose was to examine any possible effects DORA-22 may have on negative valence stimuli-induced arousal and compare it with at least one of the GABA-A receptor modulators. We were able to confirm that triazolam was suitable for this purpose. Nevertheless, we admit that possible species difference in the effectiveness of different drugs is a limitation of this study.

The prolonged latency to wake up during triazolam treatment did not seem to be caused by a general inhibition of waking systems in the brain because there was no significant change in the latency to wake in response to hypoxic stimulus (Figure 2D). Rather, it seemed to be caused by inhibition of sensory input pathways. An increase in latency to wake from triazolam treatment in the auditory and vestibular stimuli tests indicated an importance of the thalamus where the GABA-A receptor is involved in sensory gating before the signal reaches the cerebral cortex (McCormick and Bal, 1994). The unchanged latency to wake in response to hypoxic stimulus may be explained by the fact that autonomic reflex-like responses do not depend on the thalamo-cortical pathway. A possible contribution of the medullary GABAergic pathway, however, has been proposed to be responsible for arousal in response to intermittent hypoxia (Darnall et al., 2012). One of the more surprising results was that the latency to wake in response to olfactory stimulus was also prolonged with triazolam treatment. It is interesting because olfactory information directly reaches the cerebral olfactory cortex from the glomerulus in the olfactory bulb and thus is not gated by the thalamus. The periglomerular cells of the olfactory system that contain the GABA-A receptor (Panzanelli et al., 2007) may be responsible for the gating effect.

We noticed that latency to return to sleep after arousal by aversive stimuli (150–300 s in vehicle and 50–100 s in DORA-22 and triazolam, Figures 2A–C) was similar to and did not exceed awake episode durations in our tests where no stimuli were given (~300 s in vehicle and ~100 s in DORA-22 and triazolam, Figure 1F). This result indicates that the aversive stimuli used in this experiment were mild enough to not elicit a continuous alerting effect on the mice. Due to the observation that DORA-22 and triazolam were effective for approximately 4 h, at least without stimulation (Figure 1), and aversive stimulus-induced arousal testing was performed within this time window, it was not surprising to observe a short latency to return to sleep in the drug-treated groups.

We used within-subject design to compare drugs’ effect for each aversive stimulus. This design gives us higher statistical power than independent design but at the same time possible habituation effect to the stimulus may distort the results. However, such habituation effect seemed minimal in the current experimental setting and randomized order of dosing. If the habituation effect took place, then some animals that were treated with vehicle after DORA-22 and/or triazolam should had longer latency to arousal than those that was treated with vehicle as the first drug. This seemed not the case since the data distribution for vehicle showed very narrow range (Figure 2). In addition to statistical high power, within-subject design needs fewer animals than independent design. Therefore, when habituation effect is enough smaller than the effect of interest, within-subject design is a good choice of experimental design.

Orexin exerts its wake promoting/stabilizing effect through activation of monoaminergic systems such as noradrenaline in the locus coeruleus, serotonin in the dorsal raphe, and histamine in the tuberomammillary nucleus (Sakurai, 2007). Thus, the sleep-promoting effect of orexin receptor blockade is believed to be elicited by an inhibition of orexin signaling in these nuclei. Although orexin receptors are also expressed in some thalamic nuclei (Marcus et al., 2001), possible effects of orexin receptor blockade on sensory input gating have not yet been reported. It is of interest to note that orexin is involved in stress-induced analgesia (Watanabe et al., 2005; Inutsuka et al., 2016) as well through possible activation of descending pain-inhibitory pathways (Ho et al., 2011). If orexin does also plays a role in sensory input gating, it seems plausible for it to also be anti-nociceptive. Therefore, any effect that orexin receptor blockade might have on sensory gating may occur in a direction opposite to that of any sleep-related sensory gating.

## Conclusion

The findings of this study are consistent with the distinct mechanisms of these sleep promoting therapies; GABA-A receptor activation by triazolam is thought to induce widespread CNS suppression, which includes sensory gating systems, while DORA-22 more specifically targets sleep/wake pathways through orexin receptor antagonism. These data support the notion that DORA-22 preserves the ability to wake in response to aversive and consciousness-inducing stimuli, regardless of modality, while remaining effective in the absence of the threat. This study provides a unique and important point of view for the evaluation of the safety of these hypnotics.

## Author Contributions

SI and TK designed the study. All the authors conducted the study and analyzed the data. SI and TK wrote the manuscript. All authors approved the final version of the manuscript.

## Conflict of Interest Statement

The authors declare that the research was conducted in the absence of any commercial or financial relationships that could be construed as a potential conflict of interest.

## Abbreviations

CNS: central nervous system
DORA: dual orexin receptor antagonist.
